# Bioleaching as a biotechnological tool for metal recovery: from sewage to space mining

**DOI:** 10.3389/fbioe.2025.1712157

**Published:** 2026-01-15

**Authors:** Archana Paimpillil Abraham, Simone Schopf

**Affiliations:** Brandenburg University of Technology Cottbus-Senftenberg, Cottbus, Germany

**Keywords:** bioleaching, biomining, metal recovery, space mining, sustainable technology

## Abstract

Heavy metals are essential for technological and economic growth but can cause serious environmental and health problems due to their toxicity and persistence. Traditional methods for metal recovery often have high costs and can create secondary pollution. Bioleaching offers a sustainable, low-energy, and eco-friendly alternative, effectively recovering metals from low-grade ores and various waste materials. Recovering metals from secondary sources such as industrial and electronic waste reduces the need for new mining, thus conserving natural resources and supporting circular economic goals. Recently, biomining has expanded beyond Earth, showing promising results in space environments. This review discusses the current understanding of bioleaching processes, their potential for sustainable metal recovery on Earth and in space, their challenges, and future perspectives. Overcoming technical challenges, such as raw material composition, slow reaction kinetics, optimization of process parameters, and addressing safety concerns is crucial. A further increase in research focus aiming at scaling up bioleaching technology is essential, alongside addressing ethical and economic concerns related to space mining.

## 1 Introduction

Metallic materials are inorganic substances, usually combinations of metallic elements, such as iron, titanium, aluminum and gold, which may also contain small amounts of non-metallic elements, such as carbon, nitrogen, and oxygen (Minay and Boccaccini, 2005). Metals are fundamental to modern society and sustain global economic activities (Zhang T. et al., 2025). While technological advancements and increased industrial and agricultural productivity are crucial for the global economy, they have also contributed to the release of toxic metals impacting public wellbeing and the environment (Jeyakumar et al., 2023; Xu L. et al., 2024; Xu W. et al., 2024). Heavy metals and metalloids are typically defined as elements with a density greater than 5 g/cm^3^. This group includes a broad spectrum of elements such as antimony, arsenic, asbestos, cadmium, chromium, copper, lead, manganese, mercury, molybdenum, nickel, selenium, thallium and zinc, among others (Adnan et al., 2024; Priyanka et al., 2024; Han Y. et al., 2025). Heavy metals exhibit toxicity even at trace concentrations (Abdel-Rahman, 2022; Piwowarska et al., 2024). Elements such as arsenic, cadmium, chromium, copper, lead, mercury, nickel and zinc are recognized as environmental pollutants due to their toxicity and tendency to bioaccumulate within food chains, ultimately threatening both ecosystem integrity and human health (Sweta and Singh, 2024; Meftah et al., 2025).

Anthropogenic activities such as mineral extraction, industrial emissions, agricultural runoff, and waste management (Xu W. et al., 2024; Meftah et al., 2025) are primary contributors to metal pollution whereas natural processes such as rock weathering, leaching, soil erosion and volcanic activity play a relatively minor role (Piwowarska et al., 2024). Among these, mineral extraction has been reported to release heavy metals such as antimony, arsenic, cadmium, chromium, copper, lead, selenium, thallium, and vanadium (Khosaravi et al., 2020; Ren et al., 2022), while industrial processes contribute arsenic, cadmium, chromium, copper, lead, manganese, mercury, nickel, and zinc (Huang et al., 2019; Chen et al., 2025; Khan et al., 2025). Cadmium, copper, lead, and zinc have also been detected in agricultural runoff (Chheang et al., 2021), whereas landfills have been reported to cause contamination involving cadmium, chromium, iron, nickel, lead, and manganese (Paoli et al., 2012; Wu et al., 2022; Drall et al., 2025).

Sewage sludge derived from wastewater (Mohamed et al., 2023), acid mine drainage (AMD) (Sweta and Singh, 2024) and hazardous electronic waste (e-waste) (Sandwal et al., 2025) are other typical sources of heavy metals. Sewage sludge contains heavy metals such as antimony, arsenic, cadmium, chromium, copper, iron, lead, manganese, mercury, nickel, and zinc (Świerczek et al., 2021; Mohamed et al., 2023). AMD is characterized by the presence of arsenic, cadmium, cobalt, chromium, copper, lead, mercury, molybdenum, nickel, and zinc (Qin et al., 2024; Wilfong et al., 2024). E-waste consists of arsenic, cadmium, chromium, copper, lead, mercury, nickel, selenium, and zinc (Adu and Aneke, 2025). A comprehensive review of heavy metal pollution in riverine sediments from various Asian and European countries indicated moderate contamination levels (1 ≤ Contamination Factor <3) throughout the geological units, excluding lead, cadmium and copper (Zeb et al., 2024). Therefore, the removal of these toxic metals from the environment is a critical necessity (Sarkodie et al., 2022). On the other hand, metals are increasingly recognized as sustainable resources due to their high recyclability potential which helps to lower the carbon footprint associated with the end products (Rendón-Castrillón et al., 2023).

Conventional methods of metal extraction such as hydrometallurgical processes, pyrometallurgical processes, or a combination of both, are efficient and fast. However, these methods have several disadvantages, including the requirement for high-grade raw materials, secondary pollution, high energy costs and the emission of toxic gases (Pathak et al., 2022; Sarkodie et al., 2022; Xia and Ghahreman, 2023; Madhavan et al., 2024; Nkuna and Matambo, 2024; Saim and Darteh, 2024; Wu et al., 2024). The bioprocessing of mineral ores and enriched extracts for the recovery of rare earth elements (REE) and other metals is a well-established and continually advancing field within biotechnology (Hussien, 2025). In contrast to conventional techniques, bioleaching is recognized as an economical and energy-efficient alternative that offers a streamlined process without requiring specialized equipment (Nkuna and Matambo, 2024). While ‘bioleaching’ refers to the extraction of metal cations from often nearly insoluble minerals in ores through biological processes such as acidification, oxidation and complexation, ‘biomining’ encompasses both bioleaching and bio-oxidation applications (Vera et al., 2022; Cozma et al., 2024). Bioleaching is used as a preliminary metal extraction technology from ores and has gained popularity in the mining industry due to its cost-effective metal recovery (Rendón-Castrillón et al., 2023; Jia et al., 2024).

**FIGURE 1:**
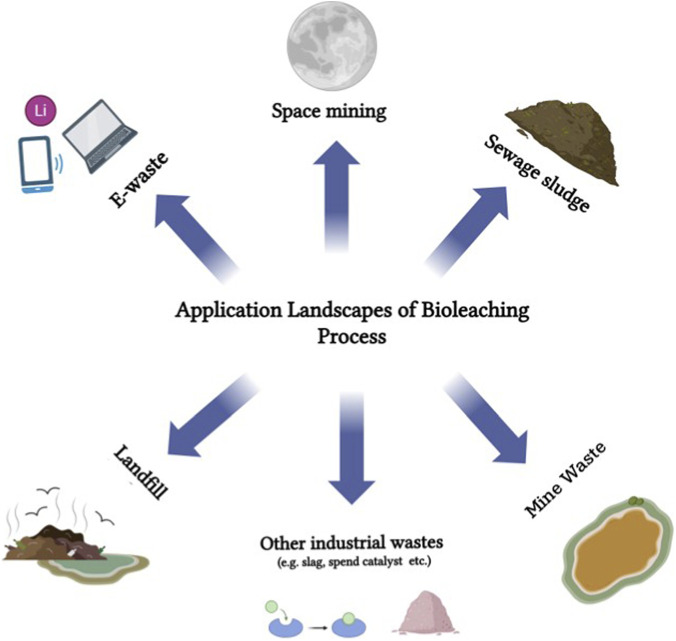
Application landscapes of bioleaching process. From domestic sewage to space mining, bioleaching presents an effective biotechnological tool for metal recovery across diverse sectors.

The identification of *Thiobacillus ferrooxidans* and its ability to accelerate the pyrite oxidation and subsequent dissolution marked the beginning of the biomining era (Colmer et al., 1950; Kelly and Wood, 2000; Johnson, 2018). Dump leaching of run-of-mine copper waste rocks was carried out in the mid-1960s at mines operated by the (then) Noranda corporation in the United States (Johnson, 2018). In the 1970s, *in situ* bioleaching was employed in Canada to recover residual uranium from depleted mines (Wadden and Gallant, 1985). Ever since its potential and advantages were identified, bioleaching has paved the way for widespread applications and innovations. Beyond the mining sector, other explored areas include sewage sludge (Shokoohi et al., 2025), electronic waste (Dalal Guin and Deswal, 2024), fly ash (Lisafitri et al., 2025), tailings (Liao et al., 2021), slag (Pirsaheb et al., 2021), spent catalysts (Qian et al., 2020) and red mud (Han Z. et al., 2024), to name a few. Additionally, biomining experiments have also been conducted in space to explore the potential for metal extraction from celestial bodies (Santomartino et al., 2020). Figure 1 illustrates various practical applications of the bioleaching process for metal recovery.

**FIGURE 2:**
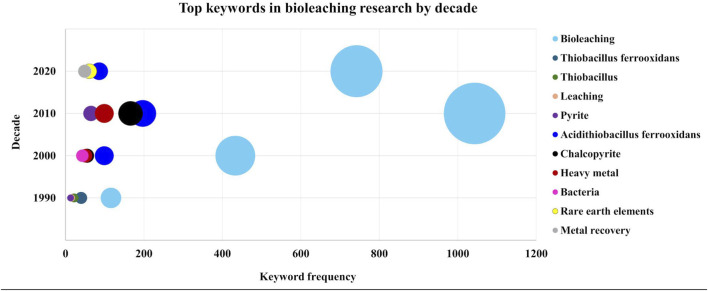
Evolution of research trends in bioleaching: a keyword analysis (1970 -2025). Keyword analysis helps to understand the transition in research focus over the decades. It indicates a growing interest in metal recovery in recent years compared to previous decades.

To better understand the evolution of research interests within the field of bioleaching, a timeline diagram (Figure 2) was created based on keyword analysis from the Web of Science database (data collected up to 9 May 2025). Articles published in scientific journals between 1970 and 2025 were filtered using the term ‘bioleaching’. For each decade, the top five most frequently cited keywords were identified, with the circle diameter proportional to keyword frequency. The diagram highlights the changing significance of keywords over time and captures key developments in the field, such as the renaming of *Thiobacillus ferrooxidans* to *Acidithiobacillus ferrooxidans (A. ferrooxidans)*. It also shows the consistent prominence of chalcopyrite - a key copper ore - in bioleaching research, alongside an increasing focus on metal recovery in recent years, reflecting a growing awareness of environmentally driven innovation.

Bioleaching has been the subject of increasing research over recent decades. However, there is a lack of comprehensive reviews examining its potential applications across diverse waste streams, including space mining. This paper aims to provide an extensive review of recent scientific advancements in bioleaching applied to various waste types, such as sewage sludge, industrial waste, landfills, mine waste and electronic waste. It also briefly explores the underlying mechanisms and potential areas for optimization. Bioleaching case studies for different waste streams are presented alongside gaps in the current literature. Bioleaching efforts for extraction of REE and its potential application in extra-territorial frontiers, such as space mining, are also discussed. The study concludes with a discussion of the present challenges and future considerations of bioleaching, emphasizing its potential role in both terrestrial and extraterrestrial metal recovery efforts.

## 2 Mechanisms of bioleaching

Extensive research has already been conducted on the detailed mechanism of bioleaching (Li J. et al., 2022; Sarkodie et al., 2022; Vera et al., 2022; Jones and Santini, 2023; Tezyapar Kara et al., 2023; Hussien, 2025). Microorganisms are essential for extracting metals from ores, especially those rich in sulfides (Hussien, 2025). Several process characteristics, including pH, temperature, the type of carbon source, carbon supply and oxygen availability, significantly affect the activity of microorganisms in bioleaching processes (Naseri et al., 2023). Acidophilic bacteria and archaea, which thrive in bioleaching environments, can metabolize iron and reduced inorganic sulfur compounds, thus facilitating the dissolution of minerals (Vera et al., 2022). This section provides a brief overview of the fundamentals of bioleaching.

Bioleaching can occur *via* two modes: 1) direct bioleaching and 2) indirect bioleaching. Commonly, in direct bioleaching, microorganisms bind to the mineral surface and oxidize sulfides directly without relying on ferric iron (Fe^3+^) as an oxidizing agent. However, Fe^3+^ can also be involved in direct bioleaching when it is captured with glucuronic acids within the extracellular polymeric substances (EPS) (Xu J. et al., 2022). Conversely, in indirect bioleaching, ferrous iron (Fe^2+^) undergoes biological oxidation to Fe^3+^, which then serves as the chemical agent that oxidizes the metal sulfides (MS) (Vera et al., 2022; Jones and Santini, 2023). The sulfide dissolution rate is 100–1,000 times higher in the presence of Fe^3+^ compared to dissolved oxygen under acidic conditions, with the oxidation of sulfide minerals by Fe^3+^ being the rate-limiting step in the bioleaching process (Li J. et al., 2022).

The different types of bioleaching are 1) oxidative bioleaching, 2) acid bioleaching, and 3) reductive bioleaching (Vera et al., 2022).

### 2.1 Oxidative bioleaching

**FIGURE 3:**
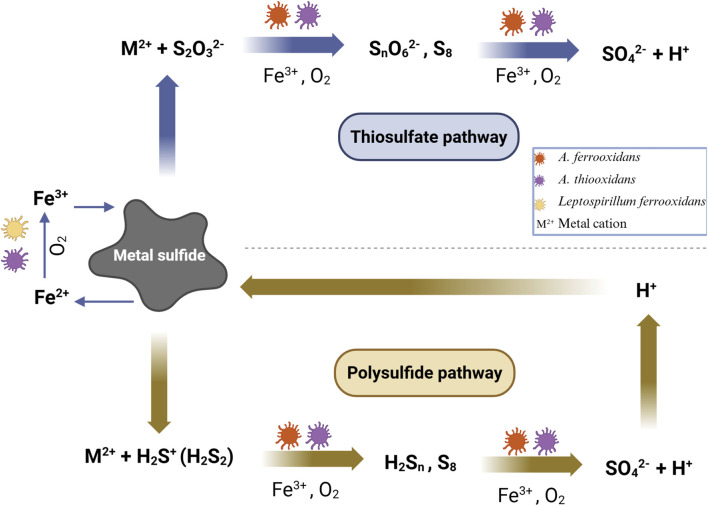
Schematic comparison of thiosulfate and polysulfide pathways in bioleaching (Rohwerder et al., 2003; Vera et al., 2022; Tezyapar Kara et al., 2023). In the thiosulfate pathway, Fe^3+^ ions reduce to Fe^2+^, releasing metal cations and thiosulfate, which further oxidizes to sulfuric acid. In the polysulfide pathway, protons react with MS, yielding metal cations and polysulfides, with primary reaction byproduct being elemental sulfur forming in the absence of sulfur-oxidizing bacteria.

In oxidative bioleaching, microorganisms break down MS, employing O_2_ as the electron acceptor. Chemical reactions in oxidative bioleaching involve the thiosulfate and the polysulfide pathways (Figure 3). In industry, oxidative bioleaching serves as a method for recovering metals from sulfide-based minerals (Vera et al., 2022). In the thiosulfate pathway, Fe^3+^ ions target MS by removing electrons, resulting in their reduction to Fe^2+^ ions. Consequently, the MS mineral discharges metal cations (M^2+^) along with water-soluble intermediate sulfur compounds (thiosulfate intermediates, S_2_O_3_^2-^). The S_2_O_3_^2-^ intermediates undergo further oxidation, either abiotically or biotically by sulfur-oxidizing bacteria such as *A. ferrooxidans* and *Acidithiobacillus thiooxidans* (*A. thiooxidans*). The product of this oxidation is sulfuric acid. Bacteria that oxidize Fe^2+^, like *A. ferrooxidans* and *L. ferrooxidans* (*Leptospirillum ferrooxidans*) facilitate the re-oxidation of Fe^2+^ to Fe^3+^ ions in acidic conditions. In the polysulfide pathway, protons initiate an additional reaction with the valence electrons of MS. Subsequently, the MS mineral releases M^2+^ and polysulfide (S_n_^2-^) intermediates. Resulting sulfur compounds undergo oxidation both biotically (by sulfur-oxidizing bacteria) and abiotically. The primary reaction byproduct, which builds up in the absence of sulfur-oxidizing bacteria is elemental sulfur (Roberto and Schippers, 2022; Vera et al., 2022).

### 2.2 Acid bioleaching

Acid bioleaching combines acid production and oxidation, with sulfur-oxidizing bacteria generating sulfuric acid to dissolve metals (Vera et al., 2022). The acid bioleaching process can be classified into two distinct categories - irrigation leaching and stirred-tank leaching (Whitworth et al., 2022). Irrigation leaching includes heap, dump, and *in-situ* leaching, where an acidic solution percolates through crushed ore (Pradhan et al., 2008). Heap leaching involves stacking crushed ore on an impermeable pad and applying a leach solution that promotes mineral dissolution through the activity of microbes producing Fe^3+^ and H_2_SO_4_ (Whitworth et al., 2022). In dump leaching, waste rock undergoes treatment directly at locations where it is discarded, whereas *in-situ* leaching focuses on minor deposits and ores with low mineral content within sites that are either abandoned or situated underground (Pradhan et al., 2008).

Stirred-tank leaching employs a series of continuous-flow reactors that are either highly aerated or anaerobic, depending on if the goal is bio-oxidation or anaerobic mineral dissolution (Whitworth et al., 2022). Ore or mineral concentrate processed to a fine particle size, along with nutrients like NH_4_^+^ and PO_4_^3-^, move sequentially through the tanks, facilitating metal solubilization (Rawlings, 2005). Although this method is more costly, it is preferred for extracting high-value metals such as gold because it achieves metal dissolution much faster than heap leaching (Rawlings, 2005). Microorganisms in these systems break down the sulfidic mineral matrix, ultimately enabling gold solubilization through cyanide treatment (Komnitsas and Pooley, 1989).

### 2.3 Reductive bioleaching

Reductive bioleaching involves the microorganisms-catalyzed dissolution of ore or solid materials through chemical reduction reactions (Roberto and Schippers, 2022). In reductive bioleaching, the dissimilatory reduction of Fe^3+^ to Fe^2+^ by acidophilic bacteria occurs in oxygen-free or low-oxygen environments. Bacteria utilize either inorganic electron donors such as elemental sulfur and hydrogen or organic electron donors, like glucose, to reduce Fe^3+^. Through reductive bioleaching, nickel, cobalt, manganese, and copper can be extracted from oxidized ores. It is also employed to remove Fe^3+^ coatings from REE-bearing minerals, and to enhance their extraction (Johnson and du Plessis, 2015).

Reductive bioleaching of limonitic laterites using *A. ferrooxidans* has been demonstrated in laboratory settings and is known as the Ferredox process. The Ferredox method is suggested for the treatment of limonitic laterite ores to recover cobalt and nickel through anaerobic reductive dissolution using autotrophic acidophilic bacteria. The reductive bioleaching technology could improve metal recovery from active mines and convert untouched ores, limonite reserves, and tailings from laterite ore processing into valuable resources (Roberto and Schippers, 2022).

Reductive bioleaching is documented to be seven times more efficient than oxidative bioleaching (Johnson and du Plessis, 2015). Tetrathionate hydrolase is identified as a crucial enzyme in the reductive bioleaching pathway. This enzyme facilitates the breakdown of tetrathionate into elemental sulfur, sulfite, trithionate, and pentathionate, which are subsequently fully oxidized to sulfate in solution (Brar et al., 2021).

The bioleaching process offers several advantages over conventional metal leaching techniques. It is environmentally friendly, requiring less energy and thus lowering costs, having simpler operation, minimizing dependance on skilled labor, generating fewer toxic gases, and reducing secondary pollution (Biswal and Balasubramanian, 2023; Dong et al., 2023; Hussien, 2025). The following sections explore in detail the feasibility of bioleaching applications for extracting metals from various waste streams and space mining.

## 3 Multisectoral applications of bioleaching

### 3.1 Sewage sludge

Sewage sludge, generated during the biological treatment of wastewater (Molaey et al., 2024), is a potential reservoir for metal recovery. Surface runoff, sewer infrastructure degradation, and the discharge of industrial effluents into municipal treatment systems are the main causes of the buildup of toxic metals in sewage sludge (Siddiqui et al., 2023). Applying the metal-contaminated sludge to land often leads to the release of these metals into soil, facilitated by soil microbes that decompose the organic matter within the sludge. Metals have been shown to accumulate over time, resulting in the inhibition of soil microorganisms. A consequence thereof is the reduction in beneficial bacteria that participate in critical processes like nutrient cycling, nitrogen fixation and organic matter decomposition (Dhanker et al., 2021; Molaey et al., 2024). When contaminated plant material is ingested, it can accumulate in human tissues over time, leading to disruptions in vital bodily functions (Ilyas et al., 2024). To maintain soil quality and integrity of agricultural products, global regulations have imposed limits on the application of sewage sludge (Table 1).

**TABLE 1:** Typical heavy metal concentrations in sewage sludge and limit values for agricultural use (Fijalkowski et al., 2017; Kaur and Garg, 2021; Shokoohi et al., 2025).

Heavy metal	Concentration (mg/kg of dry matter)	Limit value (mg/kg of dry matter) directive 86/278/eec
Ag	1.1–14.7	Not limited
As	5.6–56.1	Not limited
Cd	0.3–12	20–40
Co	1.5–16.7	Not limited
Cr	10.8–1,542.2	Not limited
Cu	27.3–3,323	1,000–1750
Mo	1.7–12.5	Not limited
Mn	75.2–959.7	Not limited
Ni	8.6–422	300–400
Pb	4.0–429.8	750–1,200
Ti	65.2–1,070.9	Not limited
V	2.3–135.4	Not limited
Zn	518–1,000	2,500–4,000

In contrast to organic pollutants, metal ions are characterized by their non-biodegradability and require a wide range of treatment methods such as thermal treatment, electrodialysis, bioleaching, vermicomposting, biosurfactant usage, constructed wetlands, acid and chemical leaching, chelation, ion exchange, and chemical precipitation (Shokoohi et al., 2025). Among the available methodologies, bioleaching - encompassing both mixed and single cultures - has been identified as both convenient and efficient (Zhao et al., 2024). In research focused on bioleaching sewage sludge, *A. thiooxidans* and *A. ferrooxidans* are the most frequently cited acidophiles (Gonçalves et al., 2023). However, studies have also reported other microbial species. Several studies examining microbial community composition during bioleaching have reported the presence of *Acidithiobacillus caldus*, *Acidophilium*, *Leptospirilum,* and *Nirospira* within the sludge (Pathak et al., 2009). At pH levels below 2, *A. caldus* has been reported as the primary bacterium in bioleaching, replacing *A. thiooxidans*, which was previously thought to be the dominant acidophile in sludge bioleaching (Bouchez et al., 2006). Xiao et al. (2019) studied bioleaching of sewage sludge involving an electrochemical pretreatment step. Although *A. thiooxidans* and *A. ferrooxidans* were utilized in the biological treatment phase, next-generation sequencing analysis indicated the presence of *Acidocella*, *Alicyclobacillus* spp., *Nitrospira, Rhodanobacter* as well as *Thiobacterales* as the most abundant bacterial groups involved in the process (Xiao et al., 2019). In a study by Wong and Gu (2004), co-inoculation of *A. ferrooxidans* ANYL-1 with *Blastoschizomyces capitatus* Y5 enhanced heavy metal solubilization and reduced the bioleaching time for chromium, copper, and zinc by approximately 50% compared to treatment with *A. ferrooxidans* ANYL-1 alone (Wong and Gu, 2004).

Bioleaching has recently been recognized as a promising pre-treatment strategy for the dewatering of excess sludge, with its effectiveness making it a viable alternative to conventional chemical and physical methods (do Nascimento et al., 2022). EPS located on or adjacent to sludge surfaces can hold significant quantities of water through hydrogen bonds or electrostatic interactions (Neyens et al., 2004). An excess of EPS contributes to poor sludge dewaterability, as its gel-like structure limits the passage of water through sludge floc pores. Furthermore, because EPS molecules are negatively charged, elevated levels enhance electrostatic repulsion among cells, resulting in weaker floc formation and impaired settling performance (Mowla et al., 2013). Hence, releasing EPS-bound water is crucial for improving sludge dewatering efficiency. During bioleaching, bacterial oxidation of the substrate progressively acidifies the medium, breaking sludge flocs and neutralizing the negative surface charges of the particles. This decreases repulsive forces among the particles, thereby promoting floc aggregation and facilitating the release of entrapped water, which improves dewaterability (Kurade et al., 2016). Sludge dewaterability is commonly evaluated using Capillary Suction Time, Time to Filter, and Specific Filtration Resistance, and a decrease in these values reflects improved dewatering performance (do Nascimento et al., 2022). There have been several research studies carried out that demonstrate metal recovery from sewage sludge through bioleaching. In the research undertaken by Shokoohi et al. (2025), bioleaching was employed to extract various heavy metals from sewage sludge. An inoculum enriched with sludge and iron-oxidizing bacteria, containing 2 g ferrous sulfate heptahydrate (FeSO_4_ x 7H_2_O), was utilized to recover metals from sewage sludge collected from a municipal wastewater treatment facility. The concentration of heavy metals continuously decreased throughout a span of 15 days. The removal efficiency achieved levels of 62.7% for aluminium, 80.7% for copper, 43% for lead, and 75.5% for zinc. The findings further demonstrated a 66.87% decrease in the sludge’s specific filtration resistance, thereby enhancing its dewaterability. In a separate study conducted by Zhang X. et al. (2022), *A. thiooxidans* was used to oxidize sulfur, resulting in the acidification of sludge derived from wastewater treatment facilities. Researchers compared two treatment schemes: (1) simultaneous aerobic digestion with bioleaching and (2) aerobic digestion as a pre-treatment followed by bioleaching. It was found that scheme (1) yielded better results, with removal rates of zinc, copper, manganese, and nickel of 87.9%, 63.3%, 69.3%, and 58.2%, respectively. Furthermore, it exhibited a superior sludge-reduction effect, achieving reductions of 54.0% and 64.8% in mixed liquor suspended solids and mixed liquor volatile suspended solids respectively. However, each treatment scheme improved sludge dewatering performance.

It is important to note that bioleaching processes utilizing *in-situ* iron-oxidizing microorganisms generally do not necessitate the rigorous control and continuous monitoring typically needed in laboratory conditions. Despite the clear benefits of this process in comparison to other aerobic bioleaching methods, the substantial consumption of Fe^2+^ ions continues to be a major limitation (Molaey et al., 2024). Additionally, the sludge characteristics play a crucial role in bioleaching efficiency of the sewage sludge. An increase in solid content has been shown to enhance the sludge’s buffering capacity, thereby reducing the rapid drop in pH and subsequently extending the bioleaching process (Yang W. et al., 2020). These findings suggest a promising avenue for further research, with the potential to optimize the process through continued investigation.

### 3.2 Landfills

Landfilling is a widely used method for municipal solid waste (MSW) disposal, known for its ease of operation and minimal investment requirements - it represents 70% of total waste management (Huang et al., 2024). Globally, MSW incineration is widely used to manage large quantities of waste (average value: 130 t/year), effectively reducing waste mass by 70% and volume by 90% (Luo et al., 2019; Gomes et al., 2020). Incineration residues - comprising fly ash, bottom ash, and various slags - are often disposed of in landfills (Luo et al., 2019). These residues present environmental concerns because they are unstable and contain elevated levels of heavy metals and toxic organic pollutants, including dioxins (Yuan and Zoungrana, 2025). Table 2 summarizes the concentrations of heavy metals detected in MSW incineration bottom ash samples in different countries.

**TABLE 2:** Composition of heavy metal concentration in MSW incineration bottom ash samples (Karyappa et al., 2025).

Country		China	Denmark	Germany	Austria	Sweden	Japan	Belgium
Heavy metal content (mg/kg)	Pb	603–3,502	6,080	13,000	4,900	4,800	3,595	1,603
	Zn	1,532–37,383	34,460	3,900	20,700	25,000	14,819	133
	Cr	79–864	125	463	245	520	119	23
	As	24–150	—	—	37	1,000	24	—
	Cd	55–562	279	373	347	240	225	2
	Cu	250–5,082	1,080	7,200	1,010	2,200	1,059	30
	Ni	19–1,584	35	122	59	92	36	41
	Hg	1–13	—	—	13	1	—	—
	Ba	147–14,000	—	1,500	927	14,000	927	147
	Mn	560–1,093	472	—	—	—	—	—
	Se	4–8	—	—	—	—	—	—

MSW incineration residues could serve as a valuable secondary resource for the recovery of critical metals. Research has indicated that bioleaching is effective in metal extraction from these residues (Gomes et al., 2020). Auerbach et al. (2019) demonstrated that incineration slags could be successfully bioleached using various bacterial strains. *A. ferrooxidans* and *L. ferrooxidans* exhibited the highest leaching efficiencies, with yields reaching 82% for aluminium and 94% for copper using *A. ferrooxidans*, and 98% for zinc using *L. ferrooxidans*. *Leptospirillum ferrooxidans* also leached REEs, achieving a 100% yield for erbium. Additionally, bacterial leaching yields were found to be similar across different particle sizes (2 mm and 4 mm mesh) (Auerbach et al., 2019). Thus, despite being landfilled or used as aggregate, MSW incineration residues contain substantial amounts of marketable metals, comparable to low-grade ores, presenting an opportunity to recover critical materials through bioleaching (Gomes et al., 2020).

E-waste represents another landfill component with considerable potential for heavy metal recovery. The recycling of e-waste and extraction of valuable metals before it reaches landfills plays a critical role in sustainable environmental management (Mathivanan et al., 2025). Bioleaching has been proven to be an effective technology for recovering valuable metals from e-waste (Lalropuia et al., 2024). A detailed discussion of e-waste bioleaching is provided in Section 3.4, Electronic Waste.

A liquid by-product, known as leachate, is discharged when MSW is continuously piled in landfills. It has a high concentration of organic and inorganic chemicals, as well as other hazardous materials like heavy metals, salts, and other trace materials (Dagwar and Dutta, 2024; Ren et al., 2025). Table 3 presents the heavy metal concentrations in landfills of different ages.

**TABLE 3:** Heavy metal concentration in leachate (Al-Balushi et al., 2024; Chen et al., 2024).

Type of leachate	Young	Intermediate	Stabilized
Landfill age (in years)	<5	5–10	>10
Heavy metals (mg/L)	>2 (low-medium concentration)	<2 (low concentration)	<2 (low concentration)

The chemical and physical characteristics of landfill leachate are predominantly determined by the stages of its maturation (Kamal et al., 2022). As infiltrating water passes through waste layers, organic matter is initially broken down into carbon dioxide, water, and heat under aerobic conditions, followed by an anaerobic phase in which complex organics are converted into acids, ammonia nitrogen, and methane (Abdel-Shafy et al., 2024). Young landfills experience elevated heavy metal concentrations because of the acidic phase of anaerobic digestion of the waste, whereas landfill ageing rises pH and consequently suppresses metal solubility and leaching (Johnson et al., 1999; Öman and Junestedt, 2008; Wang and Qiao, 2024). Nevertheless, a range of regulatory measures have been implemented in various countries to monitor heavy metal contents in landfill leachate. As shown in Table 4, the leachate discharge limits across different countries and the World Health Organization are presented.

**TABLE 4:** Regulatory thresholds for heavy metals in landfill leachate (India: Environment (Protection) Rules, 1986; China National Standard GB 16889:2024; Nalladiyil et al., 2024).

Heavy metals	Discharge limits (mg/L)						
	China	India	Malaysia	Germany	Canada	Sri Lanka	World health organization
Cd	0.01	2.0	0.01	0.1	—	0.1	0.1
Cu	0.5	3.0	0.2	—	—	3	0.2
Cr	0.1	2.0	0.05	0.1	—	0.1	0.02
Ni	0.05	3.0	0.2	1	—	3	0.2
Zn	1	5.0	2	2	—	5	5
Mn	—	2	0.2	—	—	—	0.2
Fe	—	3	5	—	0.1	—	5
Pb	0.1	0.1	0.1	—	—	0.1	0.2

Whilst residual metals in landfill leachate are concerning from an environmental perspective, these metals can also be viewed as a potential secondary resource on the one hand (Williamson et al., 2020). Kamizela et al. (2021) investigated the application of sulfur and iron oxidizing bacteria to enhance metal solubility in raw landfill leachate which served as a liquid substrate collected prior to reverse osmosis treatment. Although bioleaching is applied to solid matrices such as ores, sludge, or fly ash, the authors used the term to describe a biologically driven increase in metal mobility within an already liquid leachate matrix. In their study, the leachate (90% v/v of reactor) was amended with different combinations of *A. thiooxidans*, *A. ferrooxidans*, mixed cultures, sulfuric acid, and elemental sulfur. The research demonstrated that with the sample acidification (pH 2.0) combined with sulfur addition, metal solubilization efficiencies reached 80%–90% with *A. thiooxidans* outperforming *A. ferrooxidans*, while the mixed culture showed no added benefit (Kamizela et al., 2021). Notably this study does not represent traditional solid-phase bioleaching but rather demonstrates that biological acidification can be used to mobilize metals present in liquid landfill leachate, prior to downstream treatment.

Collectively, the bioleaching of diverse landfill-associated components demonstrates the growing potential of biologically driven processes to recover valuable metals, reduce environmental risks, and contribute to more sustainable and circular approaches to landfill management.

### 3.3 Mine waste

Mining and mineral processing generate large amounts of hazardous waste materials, including ash, dust, slag, tailings, metals, chemicals, and particulate emissions, among others (Ayangbenro et al., 2018).

The most significant of these wastes is AMD (Rodríguez-Galán et al., 2019). AMD is a highly acidic and metal-laden leachate originating from mining pits, waste rocks, or tailings deposits and represents one of the most critical water pollution issues globally (Yang M. et al., 2021; Du et al., 2022). It arises from both active and abandoned mining operations, particularly coal and gold mines (Si et al., 2024). Among sulfide minerals, pyrite is one of the principal contributors to AMD formation due to the mineral’s tendency to oxidize when exposed to oxygen, water and microorganisms (Yang M. et al., 2021). Effluents from mining activities can cause serious environmental pollution, especially when they percolate through ores rich in sulfide minerals (gold, silver, copper, *etc.*), thereby generating AMD (Nordstrom, 2000; Johnson and Hallberg, 2005).

The production mechanism of AMD can be summarised by Equation 1 (Kim and Park, 2022):

$$4\text{Fe}S_{2\left(s\right)}+15O_{2\left(g\right)}+14H_{2}O_{\left(l\right)}\to \;4\text{Fe}{\left(\text{OH}\right)}_{3\left(s\right)}+8SO_{4\left(\text{aq}\right)}^{2-}+16H_{\left(\text{aq}\right)}^{+}$$ (1)

The composition of AMD is highly variable and typical concentrations of the heavy metals in AMD across different locations are given in Table 5.

**TABLE 5:** Commonly found metals and their typical concentrations in AMD.

Location	AMD composition (mg/L)	References
Pittsburgh United States	Fe: 1.851, Mn: 1.318, Zn: 0.172, Ni: 0.131, Co: 0.0541, Cu: 0.0183, Sr: 0.836, Cd: 0.00096, As: 0.00053, Pb: 0.00146, Ba: 0.00506, Cr: 0.00236, Hg: 0.00002	Wilfong et al. (2024)
Marsberg Germany	Fe: 35, Mn: 4, Zn: 2, Ni: 0.62, Co: 0.46, Cu: 85	Arif et al. (2022)
South Africa	Fe: 289, Mn: 46, Zn: 0.35, Ni: 0.51, Si: 8.57	Kabuba and Maliehe (2021)
Guizhou Province China	Fe: 117.08, Mn: 7.39, Zn: 0.22935, Ni: 0.14361, Co: 0.14854, Cu: 0.01751, Cd: 0.00123, As: 0.00209, Pb: 0.00033, Cr: 0.00365, Al: 14.03, Mo: 0.00059, Sb: 0.00005	Qin et al. (2024)

Although naturally occurring, mining activities amplifies AMD generation (Rodríguez-Galán et al., 2019). It is estimated that more than 20,000 km of streams in the USA are impacted by AMD contamination (Acharya and Kharel, 2020). Documented consequences of AMD include increased suspended particles, heavy metal mobilization, lowered pH in aquatic systems, groundwater pollution, heavy metal penetration into the food chain, bioaccumulation, and deterioration of drinking water quality (Acharya and Kharel, 2020). Exposure to water containing toxic metals can harm human and animal cells and significantly reduce the proportion of viable cells (Acharya and Kharel, 2020). Additionally, runoff from abandoned metal mines is reported to account for roughly one-fifth of all water quality goal failures in England and Wales (Byrne et al., 2012).

In the AMD environment, chemoautotrophic acidophilic microorganisms that oxidize iron and sulfur, can oxidize and leach sulfide ores. They can release heavy metal ions by promoting the dissolution of secondary oxidized minerals by the production of organic acids from carbon (Liu Y. et al., 2023). Recently, bioleaching has emerged as a promising approach for decontaminating mine waste (Hong et al., 2024). Hong et al. (2024) utilized bioleaching to treat waste rocks containing pyrite, thereby decreasing the likelihood for AMD production at the source. The bioleaching results showed that in the presence of *A. ferrooxidans*, approximately 82% of iron and sulfur were successfully extracted from pyrite waste over a period of 40 days. The study further examined the danger of subsequent AMD release of the leached wastes and discovered that the bio-passivation layer remained persistent and efficient, with merely 8 and 160 mg/L iron discharged from various samples. Li S. et al. (2011) attempted to explore the bioleaching behaviours of combined microbial cultures on poor-quality copper sulfide ore. Researchers collected and screened ten mixed cultures from various AMDs acquired from China’s sulfide mines. The findings revealed that the mixed culture derived from the Yinshan lead-zinc mine in Dexing, Jiangxi province, China, achieved the optimal copper extraction rate of 68.89% during a 24-day bioleaching period. In this study, the primary bacteria involved in bioleaching were *A. ferrooxidans*, *A. thiooxidans*, *Alicyclobacillus spp*. and *Sulfobacillus spp*. (Li S. et al., 2011). Liu et al. (2025) highlighted the bioleaching capabilities of *Acidithiobacillus ferriphilus* QBS 3, achieving a 100% leaching rate of arsenopyrite at a 0.5% pulp concentration after 18 days (Liu et al., 2025). Baker and Banfield (2003) found *Leptospirillum* species to be equally or more prevalent in bioleaching systems compared to *A. ferrooxidans* across various temperatures and pH levels. Additionally, certain archaea, such as *Ferroplasma acidiphilum* and *Ferroplasma acidarmanus*, oxidize iron in AMD environments (Baker and Banfield, 2003). Huber et al. (1989) reported that *Metallosphaera sedula*, which thrives at 74 °C and pH 2.0, can oxidize iron and sulfur, making it suitable for high-throughput industrial bioleaching (Huber et al., 1989).

Creating an optimized bioleaching environment is crucial for each mining site, considering research on native microbial populations and practical experience to implement effective leachate remediation strategies (Rebello et al., 2021). Key considerations for onsite process demonstrations include minimizing capital and operational costs, addressing engineering challenges related to processing complex and variable low-grade feedstocks at high throughput, and meeting policy and regulatory requirements. An integrated, multi-disciplinary, and multi-stakeholder approach is essential to translate these factors into practical implementation within a circular and low-carbon economy (Zinck et al., 2019). Bioleaching efficiency is influenced by a combination of factors, including throughput, pulp density, downstream separation methods, and management of non-metallic residues, as well as ore characteristics such as grade and refractoriness, and operational conditions like nutrient availability, pH, oxygen levels, and temperature (30 °C–45 °C). Process enhancements - including the use of catalytic agents, biosurfactants, light, magnetic or electric stimulation, and microbial strain modification - can further accelerate reaction kinetics and improve overall metal recovery (Wu et al., 2009; Joulian et al., 2020; Mäkinen et al., 2020; Brar et al., 2021; Godbole et al., 2024; Dash et al., 2025). However, most studies are limited by short experimental durations, lack of statistical optimization and replication, and the use of simplified matrices, highlighting the need for standardized protocols and performance metrics (Dash et al., 2025).

### 3.4 Electronic waste

**FIGURE 4:**
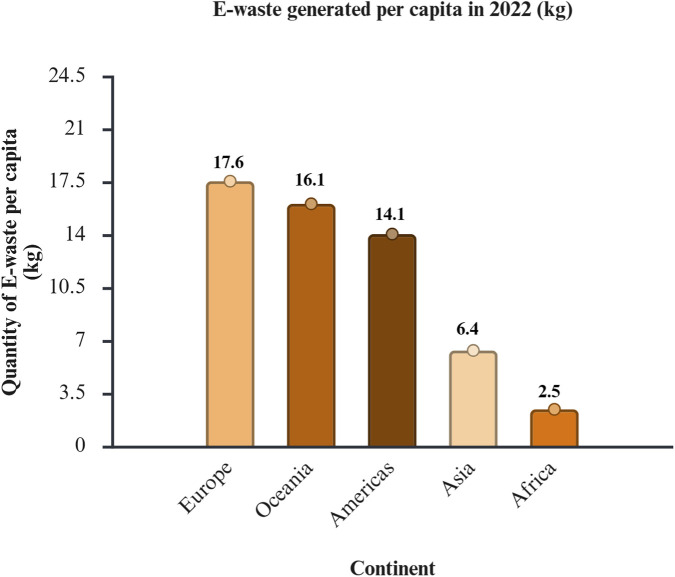
Volume of e-waste produced *per capita* in the year 2022. Data source: The Global E-waste Monitor 2024 (Baldé et al., 2024).

**FIGURE 5:**
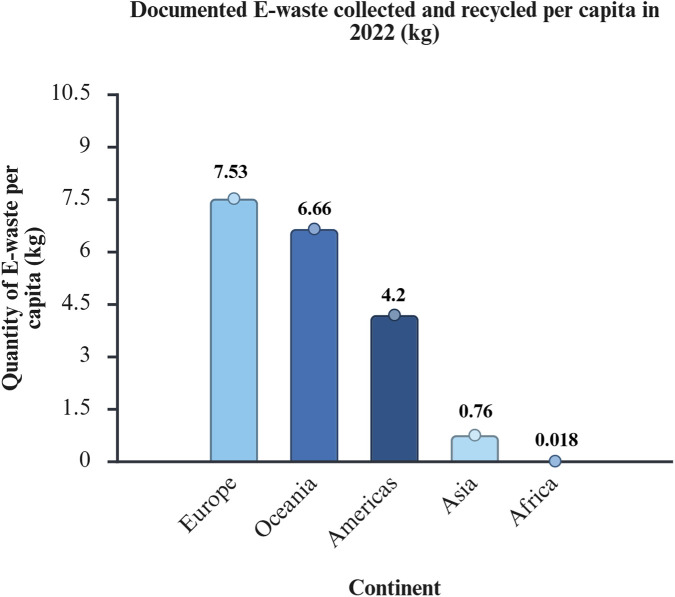
Documented volume of e-waste collected and recycled *per capita* in the year 2022. Data source: The Global E-waste Monitor 2024 (Baldé et al., 2024). The regions that generated the highest amount of e-waste *per capita* and that had the most advanced collection and recycling infrastructure were Europe, Oceania, and the Americas. African countries generated the lowest rates of e-waste but struggled to recycle it.

Electronic waste, commonly known as e-waste, is the fastest -growing waste stream worldwide (Sulaiman Zangina et al., 2023). According to Mathivanan et al. (2025), e-waste is defined as functional or damaged electrical and electronic equipment that is discarded as rubbish at a landfill. Refrigerators, information technology and telecommunications equipment, small household electrical devices, lights, and screens are some examples of e-waste (Sulaiman Zangina et al., 2023). People regularly upgrade their electronic equipment to the latest technology, resulting in the generation of unprecedented volumes of e-waste (Tipre et al., 2021). The disparity in the quantity of e-waste generation *versus* e-waste collection and recycling is depicted in Figures 4, 5.

**FIGURE 6:**
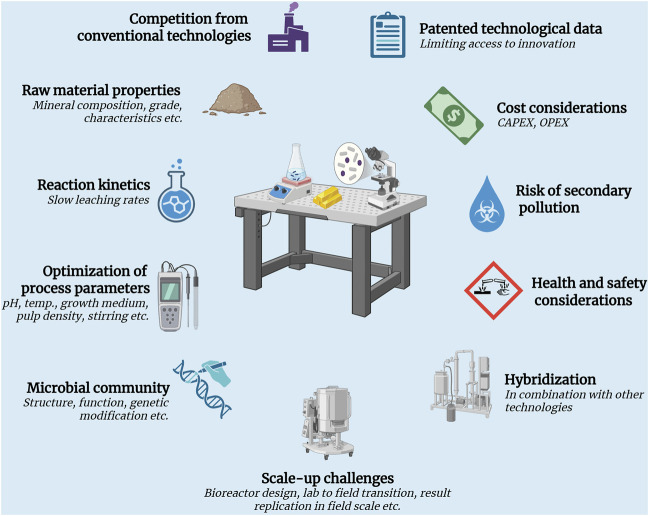
Techno-economic challenges of bioleaching technology. Right from raw material characteristics to having limited access to information on recent innovations, scaling-up of bioleaching technology faces numerous challenges. Various technical and economic obstacles must be overcome for further advancements.

Plastics, cardboard, glass, and metals are the principal components of e-waste, with heavy metals alone constituting 40% of the total composition (Harshan and Rajan, 2025). In 2022, globally generated e-waste consisted of 31 million metric tons of metals, 17 million metric tons of plastics, and 14 million metric tons of miscellaneous substances (Baldé et al., 2024). Despite raising concerns over heavy metal pollution, just 17.4% of e-waste is being recycled globally and the rest winds up in landfills, where it stays untreated (Adu and Aneke, 2025). Table 6 lists the types of heavy metals found in various forms of e-waste.

**TABLE 6:** Examples of heavy metals found in e-waste (Adu and Aneke, 2025).

Type of e-waste	Heavy metal present
Batteries	Pb, Cd, Ni, Zn
Computers and laptops	Pb, Cd, Cr, As, Cu, Zn
Lamps/lighting devices	Hg, As, Se
Phones and chargers	Pb, Hg, Cu, Ni
Television sets	Pb, Zn, Cd

Both potentially hazardous (mercury, cadmium, lead, chromium, beryllium and arsenic) as well as precious (platinum, silver, and gold) metals can be retrieved from the e-waste (Brindhadevi et al., 2023; Harshan and Rajan, 2025). Owing to the significant concentrations in e-waste, recovering precious metals from it provides cost advantages over conventional mining (Ming et al., 2025). The two primary technologies for e-waste recycling, pyrometallurgy and hydrometallurgy, have a substantial adverse environmental effect and lead to secondary pollution in the form of slags, toxic fumes, and acidic wastewater (Golzar-Ahmadi et al., 2024). Compared to traditional recovery methods, bio-recovery is environmentally friendly and energy-efficient, establishing it as a sustainable technology for extracting metals from e-waste (Hu et al., 2024).

Numerous studies have already documented the bioleaching of metals from e-waste (Hubau et al., 2020; Sikander et al., 2022; Tapia et al., 2022; Thacker et al., 2022; Zhang S. et al., 2022; Dalal Guin and Deswal, 2024; John and Gurumurthy, 2025). In a recent investigation by John and Gurumurthy (2025), *A. ferrooxidans* served as a biomining agent to extract copper from discarded printed circuit boards (PCBs). Under optimized process conditions and an inoculum load of 50 mL/L, with a treatment period of 20 days, a copper recovery rate of 86.9% was achieved. Constantin et al. (2024) utilized an acidophilic consortium for bioleaching indium from discarded liquid crystal displays (LCDs). For shredded-LCDs and powdered-LCDs, the bioleaching efficiencies *via* the mixed sulfur-iron pathway were approximately 60% and 100%, respectively. In another study conducted by Tapia et al. (2022), an acidophilic, iron-oxidizing bacterial consortium was used to recover various metals from PCB residues. Bioleaching efficiency of 69% for copper and 91% for zinc was achieved after testing the consortium’s tolerance to varying PCB concentrations. Table 7 summarizes findings from various bioleaching studies conducted on different types of e-waste.

**TABLE 7:** Various studies on bioleaching of metals from e-waste.

Sl. No.	E-Waste type	Microorganisms used	Target metals and recovery percentage (%)	References
1	PCBs	*A. ferrooxidans*	86.9% Cu	John and Gurumurthy (2025)
2	Shredded LCDs	Consortium of acidophile culture	60% In	Constantin et al. (2024)
	Powdered LCDs		100% In	
3	PCBs	*Lysinibacillus* sp. SDG4	99.7% Al 99.6% Zn 93.7% Cu 59.2% Fe	Dalal Guin and Deswal (2024)
4	PCB	*Tissierella*, *Acidiphilium* and *Leptospirillum* bacteria	69% Cu 91% Zn	Tapia et al. (2022)
5	PCB and tantalum capacitor	Consortium of *A. ferrooxidans*, *L. ferrooxidans* and *A. thiooxidans*	100% Cu 85% of each Zn, Fe, Al and Ni	Sikander et al. (2022)
6	PCBs	*Acidithiobacillus* spp.	89.1% Cu 68.5% Ni	Zhang S. et al. (2022)
7	Smartphone display	*A.ferroxidans*	100% In 5% Sr	Pourhossein et al. (2021)
8	PCBs	*Sulfobacillus benefaciens, Leptospirillum ferriphilum*	96% Cu 73% Ni 85% Zn 93% Co	Hubau et al. (2020)
9	PCBs	*A. niger* MXPE6 and *A. niger* MX5	54% Ag 2.8% Cu 0.53% Au	Díaz-Martínez et al. (2019)
10	Spent coin cells	*A. ferrooxidans*	100% Li 88% Co 20% Mn	Naseri et al. (2023)

The recent emphasis on circularity and sustainability concepts has a positively impacted the reuse and recycling of e-waste. Authorized recycling operations generate numerous job opportunities, which have a significant social impact, especially for developing countries (Ming et al., 2025). According to a life cycle study by Pokhrel et al. (2020), recycling all metals has a net positive economic impact. In PCB recycling, gold is the primary source of profit, constituting 84.13% of overall profits, while silver contributes 5.58% (Ming et al., 2025). It is worth noting that despite the unique features of REEs being crucial for future technologies, the number of patents filed for technologies related to the recovery of critical raw materials remains very limited, and that world remains remarkably dependent on the supply chains of a few countries (Baldé et al., 2024).

### 3.5 Bioleaching of rare earth elements

The REE group comprises scandium (Sc), yttrium (Y), and the fifteen lanthanide elements with atomic numbers 57 to 71: lanthanum (La), cerium (Ce), praseodymium (Pr), neodymium (Nd), promethium (Pm), samarium (Sm), europium (Eu), gadolinium (Gd), terbium (Tb), dysprosium (Dy), holmium (Ho), erbium (Er), thulium(Tm), ytterbium (Yb), and lutetium (Lu) (Dev et al., 2020; Mowafy, 2020). REEs possess distinct chemical and physical characteristics that make them indispensable in the field of high-tech manufacturing for production of materials with catalytic, electrical, luminescent, magnetic, and metallurgical properties (Vo et al., 2024).

In 2017, worldwide production of REEs reached 130,000 tons (Mwewa et al., 2022). China led this production, accounting for 85% of the output, with Australia contributing 10%, followed by Russia at 2%, India at 1%, Brazil at 1%, and smaller amounts produced in Malaysia and Vietnam (Zhou et al., 2017; Dev et al., 2020). At present, six primary ore sources are utilized for the commercial extraction of REEs: bastnesite, monazite, xenotime, apatite, loparite and ion-adsorption clays (Rasoulnia et al., 2021a). Out of which 95% of the primary REEs are sourced from bastnaesite, monazite, xenotime, and ion-adsorption clays (Owusu-Fordjour and Yang, 2023). End-of-Life products represent the secondary source of REE (Rasoulnia et al., 2021a). These include products like nickel-metal hydride (NiMH) batteries, permanent magnets, and lamp phosphors which are found in various end-of-life items, including electric and electronic devices (Binnemans et al., 2013).

Typical approaches for recovering REEs include biohydrometallurgy, pyrometallurgy, hydrometallurgy, and electrochemical techniques (Huang et al., 2025). However, hydrometallurgical, pyrometallurgical, and electrometallurgical processes, tend to generate significant secondary pollutants, such as thorium and uranium and are both chemically demanding and consume significant amounts of energy (Čížková et al., 2019). Unlike these methods, bioleaching presents an environmentally sustainable option by employing microorganisms to mobilize and recover metals, replicating natural mechanisms similar to those found in biogeochemical cycle (Gonzalez Baez et al., 2024).

The mechanism for bioleaching of REE is as follows (Dev et al., 2020; Mowafy, 2020; Vo et al., 2024): Microbial activity facilitates the solubilization of REE-bearing solid matrices (primary or secondary sources) releasing REEs. This is followed by their mobilization through three primary biochemical mechanisms: (1) The first stage, redoxolysis is a two-step process that can proceed *via* contact or non-contact processes. In contact redoxolysis, Fe^2+^ is oxidized to Fe^3+^ in the presence of oxygen, facilitated by the electron transfer from minerals to microorganisms. In non-contact redoxolysis, REEs undergo oxidative dissolution, producing REE^+^ in the aqueous phase. (2) During the second stage of acidolysis, acidic dissolution of REEs by bacteria takes place. While sulfur-oxidizing bacteria generate sulfuric acid through the oxidation of sulfides, phosphate-oxidizing bacteria facilitate REE release by liberating phosphate ions. (3) During the next stage of complexolysis, while the microbial organic acids dissolve REEs from mineral matrices, extracellular siderophores transport iron from the surrounding environment into the cell. These siderophores form complexes with REEs, facilitating their further release.

Various microorganisms, including archaea, bacteria and fungi, are employed in the recovery of REEs (Panda et al., 2021), which are capable of obtaining energy through autotrophic or heterotrophic pathways (Qu and Lian, 2013). Autotrophic microorganisms depend on carbon dioxide as their carbon source, utilize water for growth, and obtain energy from mineral ore. Among chemoautotrophic microorganisms used for REE bioleaching, *A. ferrooxidans* and *A. thiooxidans* are most reported; they efficiently oxidize ferrous ions, elemental sulfur, and thiosulfates, which subsequently contribute to sulfuric acid production (Owusu-Fordjour and Yang, 2023). Heterotrophs obtain carbon from organic sources, like glucose, to support growth and to produce various metabolites, such as amino acids, organic acids, exopolysaccharides, and proteins (Azemtsop Matanfack et al., 2022). Chemoheterotrophic fungi, particularly *Penicillium* and *Aspergillus* species, are the top reported heterotrophs in the bioleaching studies of REEs (Rasoulnia et al., 2021a). Organic acids, such as gluconic acid generated by *Aspergillus* species, perform dual roles: Firstly, they supply protons, to facilitate mineral dissolution and secondly form metal-ligand complexes, aiding their solubility in the leaching solution (Owusu-Fordjour and Yang, 2023). Table 8 summarizes selected studies on REE bioleaching from secondary sources, including the concentrations of the five most abundant REEs in each source.

**TABLE 8:** Studies on REE bioleaching from secondary sources (Dev et al., 2020; Rasoulnia et al., 2021b; Hegazy et al., 2025).

REE source	Top 5 REEs (mg/kg of dry matter)	Microorganisms aid in REE bioleaching
Phosphogypsum	Ce - 2,310, La - 1,450, Nd - 899, Pr - 235, Y - 180	*Desulfivibrio* *Acidithiobacillus, Acetobacter*
Red mud	Ce - 368, Sc - 121, La - 114, Nd - 98.6, Y - 75.7	*Penicillium tricolor* RM-10 *Aspergillus niger*
Fly ash	Ce - 195.6, La - 91.4, Nd - 88.6, Y - 62.1, Pr - 23.5	*Candida bombicola* *Phanerochaete chrysosporium*, *Cryptococcus curvatus* *A. ferrooxidans* *L. ferrooxidans*
Mine tailings	Ce - 2047, Nd - 906, La - 903, Y - 664, Pr - 239	*A. ferrooxidans* *Pseudomonas aeruginosa* *Sulfobacillus thermosulfidooxidans* *Penidiella* sp.
Magnets	Nd - 259.5, Dy - 42.1, Pr - 3.4	*A. ferrooxidans* *L. ferrooxidans*
NiMH batteries	La - 237, Ce - 67, Nd - 36	*Pseudomonas sp*. ASA235, *Gluconobacter oxydans* *Streptomyces pilosus*
Phosphor (LEDs, LCDs, and fluorescent lamps)	Y - 112.1, Ce - 4.9, Eu - 4.4, La - 3.8, Tb - 2.7	*Gluconobacter oxydans* *Komagataeibacter xylins* *Lactobacillus casei* *Yarrowia lipolytica*

Since the majority of ore minerals, including xenotime, monazite, and bastnäsite, are non-sulfidic, they lack the energy source necessary for chemoautotrophic growth, which primarily rely on sulfur or iron oxidation for energy. Consequently, chemoheterotrophic microorganisms are better suited for bioleaching of such non-sulfidic ores (Jain and Sharma, 2004). However, acidophilic autotrophs can still be employed, if an acidified cultivation medium as well as an iron and sulfur source to sustain microbial growth is supplied (Rasoulnia et al., 2021a). Also, the bioleaching by chemoheterotrophs is feasible across a pH range from neutral to alkaline, contrasting with chemoautotrophic bioleaching, which is restricted to acidic conditions (Jain and Sharma, 2004). Nevertheless, large-scale chemoheterotrophic bioleaching requires a continuous supply of organic carbon, and heavy metals in the leachate can inhibit microbial metabolism, especially in fungi due to their low and variable metal tolerance (Owusu-Fordjour and Yang, 2023). Fathollahzadeh et al. (2018) have even used a combination of both these microbial species and demonstrated an improved monazite leaching using a combination of *Enterobacter aerogenes* and *A. ferrooxidans* (Fathollahzadeh et al., 2018).

Bioleaching has proven to be an effective strategy for recovering REEs from both primary and secondary sources. Given the constrained availability of primary REE deposits, promoting REE recycling from secondary sources *via* bioleaching is crucial to ensuring a sustainable supply while minimizing the environmental impact associated with REE extraction.

### 3.6 Other industrial wastes

The ongoing disposal of industrial solid waste in the immediate vicinity results in environmental contamination and poses significant risks to public wellbeing (Adetunji and Erasmus, 2024). In addition to the waste categories discussed in previous sections, this includes ash, tailings, slag, spent catalyst, dust, sludge, red mud, phosphogypsum, *etc.* (Liao et al., 2021; Liu H. et al., 2021; Pirsaheb et al., 2021; Karim and Ting, 2022; Han Z. et al., 2024; Kinnunen et al., 2025; Lisafitri et al., 2025; Zhang J. et al., 2025).

Fly ash produced by MSW incineration can accumulate heavy metal compounds and dioxins (Wang et al., 2021; Funari et al., 2023). The environmental challenges posed by large quantities of tailings and tailings dumps are often overlooked because of the economic benefits of mining, even though they contain toxic substances in harmful quantities (Gao et al., 2021). Heavy oil fly ash, is a by-product of the combustion of heavy fuel oil used to heat boilers at power stations, contains the toxic metals vanadium and nickel (Bakkar et al., 2023).

However, these waste streams also have the potential to act as a secondary resource of strategic interest and potentially mineable metals. MSW incineration residue contains silver, antimony, cerium, lanthanum, niobium, nickel, and vanadium in the fine fractions, while gadolinium, chromium, scandium, tungsten, and yttrium are in the coarse fractions (Wang et al., 2021). Coal fly-ash, produced during burning of pulverized coal, contains REE concentrations several times richer than in coal because of the loss of organic matter during coal combustion (Bisen et al., 2025). Many mine tailings are rich in mineral-associated REEs (Gao et al., 2021). Oil sand tailings contain a high concentration of valuable metals, including vanadium, nickel, copper, titanium, zirconium, and REEs.

This indicates the possibility of metal extraction from industrial waste, which not only ensures environmental protection but also promotes circularity and sustainability. Bioleaching techniques have been successfully demonstrated in a wide variety of industries over several decades. Table 9 summarizes several research studies of metal extraction from industrial waste using bioleaching.

**TABLE 9:** Summary of bioleaching studies on various industrial wastes.

Sl. No.	Type of industrial waste	Microorganisms used	Experimental conditions				% Metal recovery	References
			pH	Temp.	Growth medium	Days		
Ash								
1	MSW Bottom Ash	*A. thiooxidans* *A. ferrooxidans*	4.0	no data	A modified 9K medium with 3 g (NH_4_)_2_SO_4_, 0.5 g K_2_HPO_4_, 0.5 g MgSO_4_·7H_2_O, 0.1 g KCl, 0.014 g Ca(NO_3_)_2_·4H_2_O and 22 g FeSO_4_·7H_2_O per L.	14	100% Cu 80% Zn 20% Pb	Funari et al. (2019)
2	Coal Fly Ash	*Bacillus toyonensi*	7.05–9.87	Room temp.	9K medium modified with 5 g glucose, and 1 g FeSO_4_ per L.	21	64% Cu 79% Zn 60% Cr 52% Ni	Lisafitri et al. (2025)
3	Heavy Oil Fly Ash	*A.ferrooxidans*	1.3	32 °C	9 K growth medium with 3 g (NH_4_)2SO_4_, 0.5 g MgSO_4_·7H_2_O, 0.5 g K_2_HPO_4_·3H_2_O, 0.1 g KCl, 0.01 Ca(NO_3_)_2_, and 44.22 g FeSO_4_·7H_2_O per L.	15	74% V 95% Ni 88% Cu	Rastegar et al. (2015)
4	Sewage Sludge Ash	*A. ferrooxidans* *A. thiooxidans*	4.4–4.7	Room temp.	no data	11	16% Fe 61% Al 41% Cu 20% Zn 13% Cr 34% Co	Zimmermann and Dott (2009)
Mine tailings								
5	Copper mine tailings	*Leptospirillum feriphilum* *Sulfobacillus* sp.*,* *Acidithiobacillus caldus* *Ferroplasma* sp.	2	45 °C	Diluted basal salts solution with 22.5 g (NH_4_)_2_SO_4_, 7.5 g Na_2_SO_4_.10H_2_O, 2.5 g KCl, 25 g MgSO_4_.7H_2_O, 2.5 g KH_2_PO_4_, 0.7 g Ca(NO_3_)_2_.4H_2_O per L.	9	84% Cu	Conić et al. (2020)
6	Lead–zinc sulfide mine tailings	*A.ferrooxidans* *Sulfobacillus thermosulfidooxidans*	1.85	30 °C	9K medium with 3.0 g (NH_4_)_2_SO_4_, 0.1 g KCl, 0.5 g K_2_HPO_4_, 0.05 g MgSO_4_·7H_2_O, 0.01 g Ca(NO_3_)_2_ per L.	16	94% Zn	Liao et al. (2021)
Slags								
7	Smelter slag	*A. thiooxidans* *A. ferrooxidans* *Leptospirillum ferriphilum*	2.0–2.2	27 °C	0.5 g (NH_4_)_2_SO_4_, 0.5 g K_2_HPO_4_, 0.5 g MgSO_4_·7H_2_O per L and trace metals.	25	83% Cu 14% Zn	Tuovinen et al. (2015)
8	Steel Slag	*A. ferrooxidans*	1.8	22 °C ± 2 °C	9 K medium consisting of two solutions - Solution A: 3 g (NH_4_)_2_SO_4_, 0.5 g K_2_HPO_4_, 0.5 g MgSO_4_.7H_2_O, 0.1 g KCl, 0.01 g/L Ca(NO_3_)_2_.4H_2_O per L. Solution B:15 g FeSO_4_.7H_2_O per L	20	59% V	Li J. et al. (2021)
9	Carbide slag	*A. thiooxidans* *A. ferrooxidans*	2	30 °C	0.5 g K_2_HPO_4_, 0.5 g MgSO_4_ ⋅7H_2_O, 2 g (NH_4_)_2_SO_4_, 0.1 g KCl, 0.01 g Ca(NO_3_)_2_, 4.468 g FeSO_4_ ⋅7H_2_O, 10 g S per L.	14	100% Zn 41.9% Ba 98.5% Ni 97.8% Li	Pirsaheb et al. (2021)
Spent catalyst								
10	Spent automotive catalyst	*Pseudomonas fluorescens*	10	30 °C	Luria-Bertani (LB)-Miller broth with 10 g tryptone, 5 g yeast extract, 10 g NaCl per L.	1	58% Pt 65% Pd 97% Rh	Karim and Ting (2022)
11	Spent Co-Mo catalyst	*Leptospirillum ferriphilum* *A. thiooxidans* *A. ferrooxidans*	4.0	32 °C ± 1.0 °C	Inorganic salt medium with 1.0 g (NH_4_)_2_SO_4_, 1.0 g KNO_3_, 1.0 g NaH_2_PO_4_, 0.5 g MgSO_4_·7H_2_O, 0.25 g CaCl_2_ per L.	8	94% Co 100% Mo	Qian et al. (2020)
Others								
12	Sulfidated Electric arc furnace dust	*Marinobacter* sp.*,* *Acidithiobacillus* sp., *Leptospirillum* sp.	2	30 °C	0 K-medium with 3.0 g (NH_4_)_2_SO_4_, 0.1 g KCl, 0.5 g K_2_HPO_4_, 0.5 g MgSO_4_ · 7H_2_O, 0.1 g Ca(NO_3_)_2_ · 4H_2_O) per L.	42	100% Zn	Kinnunen et al. (2025)
13	Theisen sludge	*A. ferrooxidans* *Acidiphilium.*	3.5–3.7	30 °C	9K25 (modified 9K medium with 25 mmol/L of sterile filtered ferrous sulfate solution of pH 1.8)	21	70% Zn 45% Cu	Klink et al. (2016)
14	Tannery sludge	*Acidophilic sulfur-oxidizing bacteria*	6	28 °C ± 1 °C	Waksman solid medium with 2.0 g (NH_4_)_2_SO_4_, 0.25 g K_2_HPO_4_, 0.25 g MgSO_4_·7H_2_O, 0.01 g Ca(NO_3_)_2_·4H_2_O, 0.1 g KCl, 22.1 g Na_2_S_2_O_3_·5H_2_O, 30 g agar powder per L. Waksman liquid medium with 0.2 g (NH_4_)_2_SO_4_, 3.0 g K_2_HPO_4_, 0.5 g MgSO_4_·7H_2_O, 0.25 g CaCl_2_·2H_2_O, 22.1 g sulfur per L.	14	95.3% Cr, 79.8% Cu, 98.9% Cd, 88.3% Pb, 99.3% Zn 97.0% Mn	Liu H. et al. (2021)
15	Red mud	*Lactobacillus pentosus*	8	30 °C	de Man, Rogosa Sharpe (MRS) medium with 10 g peptone, 10 g beef extract, 5 g yeast extract, Tween 80 1, 2 g K_2_HPO_4_, 5 g sodium acetate, 2 g diammonium citrate, 0.2 g MgSO_4_·7H_2_O, 0.05 g MnSO_4_·H_2_O per L.	7.5	31% Al	Han Z. et al. (2024)
16	Phosphogyp-sum	*Gluconobacter oxydans*	3.5	28 °C	*Acetobacter* culture medium with 0.1 g glucose, 0.02 g CaCO_3_, 0.01 g yeast extract per L.	30	55.7% Nd	Zhang J. et al. (2025)

### 3.7 Space mining

Celestial bodies, including asteroids, are commercially exhibited as holding high-grade ores of metals worth trillions in value, such as cobalt, nickel, and REEs, as well as precious metals like platinum, rhodium, osmium, iridium, palladium, and rhenium and volatile substances (Deberdt and Le Billon, 2023; Fleming et al., 2023; Vieira Neto et al., 2023). One way to enable the ongoing expansion of metal use on Earth while minimizing environmental and social impacts is to transition mining from Earth to space (Fleming et al., 2023). Space mining is increasingly framed as a techno-utopian sector that promises to open a new extractive frontier in response to the rapidly increasing need for minerals and clean power (Deberdt and Le Billon, 2023). Typical concentrations of metals found in asteroids are compared with those in Earth’s crust in Table 10.

**TABLE 10:** Typical concentrations of metals in asteroids in comparison with Earth’s crust (Hans Wedepohl, 1995; Pourmand et al., 2012; Dahl et al., 2020).

Metal concentration (mg/kg of dry matter)		
Metal	Asteroid	Earth’s crust
Fe	893,000.00	41,000.00
Co	6,000.00	20.00
Ni	93,000.00	80.00
Ru	21.50	<1
Rh	4.00	<1
Pd	16.50	<1
Os	14.50	<1
Ir	14.00	<1
Pt	29.00	1.00
Au	0.60	1.00
La	0.247	30.00
Ce	0.632	60.00
Pr	0.096	6.70
Nd	0.485	27.00
Sm	0.156	5.30
Eu	0.060	1.30
Gd	0.209	4.00
Tb	0.038	0.65
Dy	0.258	3.80
Ho	0.055	0.80
Er	0.167	2.10
Tm	0.026	0.30
Yb	0.169	2.00
Lu	0.026	0.35
Sc	5.493	16.00
Y	1.395	24.00

While the advantages of biomining, such as the need for minimal energy input, can also be employed in space (Santomartino et al., 2022), it is significantly influenced by differences in pressure, temperature, radiation, and lower gravity in space compared to Earth (Gumulya et al., 2022). Reported results show that microbes having a diameter smaller than 10 μm, such as bacteria, archaea, and various fungi, are not directly influenced by gravity (Santomartino et al., 2020; 2022). Nevertheless, the elevated radiation environment must be considered for biomining activities on the Moon, Mars or, asteroids. Some mechanisms for radiation-related stress tolerance necessitate the cell to enter a dormant state, such as through endospore production and desiccation. However, given that space mining requires metabolically active cells, additional research is crucial (Santomartino et al., 2022).

Most bioleaching mechanisms discovered thus far take place under aerobic circumstances. Nonetheless, not only that some bacteria have been shown to have anaerobic bioleaching capabilities, but also low-pressure vacuum conditions are also employed in a variety of biotechnological processes, including biomining. This demonstrates that the difficulties related to the composition and pressure of extraterrestrial atmospheres can be overcome through proper adaptation and engineering, thereby not restricting the scope of biomining applications. Indeed, low pressure may minimize the engineering requirements of bioreactors (Santomartino et al., 2022). Considering the prohibitive space temperatures and variations, as delineated in Table 11 below, any space biotechnological application must employ temperature-controlled bioreactors (Santomartino et al., 2022).

**TABLE 11:** Environmental and physical conditions of different planetary bodies (Santomartino et al., 2022).

Condition	Earth	Moon	Mars	Asteroids
Gravity	1×g	0.16 × g	0.38×g	Micro to centi-gravity
Atmosphere composition	78% N_2_, 20.9% O_2_, 0.9% Ar, 0.04% CO_2_, trace gases	Negligible	95% CO_2_, 2.8% N_2_, 2.1% Ar, trace gases	Absent
Pressure	1 bar (at sea level)	3 × 10^−15^ bar	6.1 mbar	Vacuum
Temperature	−89.2 °C to + 56.7 °C	−178 °C to + 124 °C	−153 °C to + 20 °C	Generally, < − 20 °C

Multiple biomining experiments have already been conducted in space. Among these are the European Space Agency’s BioRock and BioAsteroid experiments, which took place on the International Space Station (Santomartino et al., 2020). The BioRock experiment was to study microbe-mineral interactions in different gravity conditions with three bacterial species: *Sphingomonas desiccabilis*, *Bacillus subtilis*, and *Cupriavidus metallidurans*. It was found that all three populations grew equally well in simulated Earth gravity, Moon gravity and microgravity (Santomartino et al., 2020). Also, *S. desiccabilis* and *Bacillus subtilis* significantly increased vanadium extraction across all three gravity conditions, outperforming the sterile controls by percentages ranging from 184.92% to 283.22% (Cockell et al., 2021; Gumulya et al., 2022). The BioAsteroid experiment investigated the ability of microorganisms, specifically bacteria and fungi, to extract valuable elements from L-chondrite asteroidal material in a microgravity environment. The fungus *Penicillium simplicissimum*, considerably increased the average extraction of platinum, palladium, and additional elements from the meteoritic substrate in microgravity, outperforming non-biological leaching. These studies indicate that we do not need to mitigate different gravity variations when using these microorganisms off the Earth and biomining could in fact prove to be a great way to extract REE and valuable metals on the celestial bodies (Santomartino et al., 2020; Cowing, 2024).

While the primary goal of biomining on Mars and the Moon might be to support extraterrestrial settlements, there is currently no intention of creating a permanent human presence on an asteroid. This raises the issue of transporting the recovered minerals to their destination, suggesting that the sustainability of the process is cost-dependent and varies according to the richness of the elements and their location (Santomartino et al., 2022). Continued progress in synthetic biology, systems biology, geobiology, and process engineering may enable the development of terrestrial biomining bacteria capable of efficiently recovering metals from minerals and waste under extreme space conditions (Gumulya et al., 2022). To encourage investment in space mining research, it might be necessary to establish a cap on the environmental and social expenses incurred on Earth (Fleming et al., 2023). Furthermore, the concept of ethical mineral sourcing from outer space will increasingly challenge research and policy agendas as this new frontier is explored (Deberdt and Le Billon, 2023).

## 4 Techno-economic challenges

Although bioleaching is recognized as a technologically feasible and sustainable approach to metal extraction, several challenges must be overcome to ensure its continued development and large-scale implementation (Figure 6).

The characteristics of the raw material employed in bioleaching, such as its mineral composition, ore grade, complexity and impurity content, have a significant impact on the effectiveness of metal recovery. As reported by Gu et al. (2025), the presence of contaminants such as aluminum, sodium, iron, calcium, manganese and potassium poses a significant challenge in lithium extraction from low-grade deposits, with extraction efficiencies reaching only 32% in real brines, compared to 91% in synthetic brines. According to Harshan and Rajan (2025), e-waste presents substantial challenges for bioleaching, as current techniques are typically designed for homogeneous metal-bearing substrates. In contrast, e-waste consists of a highly complex blend of materials such as ceramic, plastic, glass and metals. Reducing particle size is generally accepted to enhance metal extraction efficiency. However, considerable energy is required for crushing and pulverization so commercially viable approaches must balance improved extraction with minimal overall energy consumption (Potysz et al., 2018).

Another technical challenge is the slow reaction kinetics of bioleaching, which is a key step in the overall process. Only few studies have explored the kinetics of bioleaching reactions, which have negatively impacted the comparability of results across various investigations (Ferreira-Filipe et al., 2025). As Owusu-Fordjour and Yang (2023) explain, the efficiency of REE bioleaching is limited by the slow kinetics of REE extraction by microorganisms. In the fungal bioleaching experiments conducted by Pathak et al. (2021), the reaction time averaged between 15 and 60 days. This is considerably longer than that of conventional hydrometallurgical and pyrometallurgical operations, reflecting the inherently slower kinetics of the bioleaching process.

To achieve optimal results from bioleaching, it is also necessary to optimize the process parameters. Nutrient deficiency, high stirring/shearing rates, heavy metal toxicity and excessive feedstock can all have a negative impact on the microorganisms during bioleaching. Additionally, an increase of the operation temperature is limited by the temperature range of the respective microorganisms (Owusu-Fordjour and Yang, 2023). Bioleaching is already applied at an industrial scale to extract copper and gold from various ores using acidophilic microorganisms. These include *A. ferrooxidans, A.thiooxidans*, *Acidithiobacillus caldus*, *Leptospirillum ferriphillum*, *Ferroplasma acidiphilum*, *L. ferrooxidans*, *Sulfobacillus thermotolerans* (Galleguillos et al., 2008; Gericke et al., 2009; Halinen et al., 2009; Demergasso et al., 2010; Soto et al., 2013). However, inadequate control of operational factors such as air flow and CO_2_ supply can cause significant shifts in pH and microbial community composition, ultimately reducing the leaching efficiency (Tezyapar Kara et al., 2023). There are also potential health and safety concerns associated with bioleaching. For example, exposure to *Aspergillus niger* spores has been linked to severe hypersensitivity reactions in humans, including asthma and allergic alveolitis. Additionally, *A. niger* is known to colonize the human body as a pathogen and could cause infections such as ear mycosis (Pathak et al., 2021).

Bioleaching technology is also being tested in combination with other technologies. Jujun et al. (2015) investigated an integrated strategy combining mechanical and biological techniques to recover copper from discarded PCBs, thereby avoiding the need for chemical treatments. Their study demonstrated that corona-electrostatic separation effectively isolates non-metallic components, thereby reducing the adverse impact of non-metal additives during bioleaching. This approach not only lowers the overall processing cost but also minimizes the risks of metal exposure to both humans and the environment. However, hybridization further complicates the process mechanism. Another concern is in relation to the substantial operational costs of the bioleaching process. Studies have identified the growth medium - primarily composed of costly carbon sources such as sucrose or glycine, along with inorganic nutrients - as the largest contributor to these expenses (Pathak et al., 2021). Another challenge relates to data access. To safeguard their innovations, many mining companies either patent their biomining technologies or may or may not make their technology available to other mining companies. However, the extent to which these technologies are shared with other industry players varies, often restricting broader access to advancements within the sector (Brierley, 2008).

## 5 Future perspectives

In light of the aforementioned challenges, it is crucial to increase research efforts to enhance our understanding of bioleaching kinetics and to accelerate progress towards industrial-scale applications, despite laboratory studies demonstrated high recovery rates for several metals (over 80% for aluminium, copper, nickel, vanadium and zinc at low pulp densities (≤5% w/v) (Tezyapar Kara et al., 2023). To address the issue of slow kinetics, future efforts should focus on developing new catalysts that promote more efficient microbial-mineral interactions (Owusu-Fordjour and Yang, 2023) or on using adapted microbial strains or pH buffers to facilitate bioleaching at higher pulp densities (Pathak et al., 2021).

Another approach is to diversify microbial consortia used in bioleaching, such as incorporating algae into bacterial and fungal co-cultures. Mixed cultures can accelerate metal solubilization and benefit from the self-sustaining nature of algae (Harshan and Rajan, 2025). However, due to the complexity of bioleaching mechanisms and the intracellular bioaccumulation of metals, the research community has not yet developed an optimized metal recovery technology for such co-cultures, which remains a key barrier to the commercialization of this approach (Dusengemungu et al., 2021; Vítová and Mezricky, 2024). Temperature optimization represents another avenue for improvement: since elevated temperatures reduce the leaching time, greater attention should be given to the use of thermophilic microorganisms (45 °C–80 °C) and to the design of mixed-culture bioreactors (Owusu-Fordjour and Yang, 2023).

In parallel, conducting life cycle assessments and techno-economic analyses will offer critical insights into the ecological footprint of bioleaching operations (Tezyapar Kara et al., 2023). To further enhance sustainability, researchers have proposed a reduction of the costs of leaching and the sustainability implications of bioleaching technology by optimizing energy sources and using alternatives, such as agricultural waste or real organic wastewater, for heterotrophs (Pathak et al., 2021; Tezyapar Kara et al., 2023).

Industrial-scale implementation will also require advancements in bioreactor design, that incorporates the development of sophisticated equipment development. For example, a dual-reactor system, with one reactor dedicated to microbial growth and metabolite (organic acid) production, and the other to the leaching operation, has been suggested as a viable configuration (Owusu-Fordjour and Yang, 2023). Finally, as bioleaching progresses towards commercialization, safety protocols that adhere to current laws and industry standards should be implemented and modified as necessary. This includes the use of appropriate personal protective equipment to mitigate risks from microbial exposure, and adequate waste storage and disposal facilities to address health and safety issues (Pathak et al., 2021).

## 6 Conclusion

While heavy metals are essential for technological and economic development, they pose significant environmental and health risks due to their toxicity, persistence and tendency to bioaccumulate. Bioleaching offers a sustainable, low-energy alternative for metal recovery, especially from low-grade ores and industrial waste. Recent advancements show its potential beyond the mining sector, with applications across various waste streams. As interest in space mining grows, biomining has emerged as a promising and energy-efficient method of extracting metals in extraterrestrial environments, with successful demonstrations at the International Space Station. These developments highlight the potential of microbial technologies to support future off-Earth resource recovery, opening a new frontier in sustainable biotechnology.
